# Comparison of Online Sexual Activity Among Iranian Individuals With and Without Substance Use Disorder: A Case-Control Study

**DOI:** 10.3389/fpsyt.2022.889528

**Published:** 2022-07-22

**Authors:** Shiva Soraya, Vahid Rashedi, Mahdieh Saeidi, Pooria Hashemi, Fatemeh Hadi, Hamidreza Ahmadkhaniha, Mohammadreza Shalbafan

**Affiliations:** ^1^Research Center for Addiction and Risky Behaviors, Psychosocial Health Research Institute, Department of Psychiatry, School of Medicine, Iran University of Medical Sciences, Tehran, Iran; ^2^Iranian Research Center on Aging, Department of Aging, University of Social Welfare and Rehabilitation Sciences, Tehran, Iran; ^3^Mental Health Research Center, Psychosocial Health Research Institute, Department of Psychiatry, School of Medicine, Iran University of Medical Sciences, Tehran, Iran; ^4^School of Medicine, Shahid Beheshti University of Medical Sciences, Tehran, Iran

**Keywords:** internet addiction, sexual behavior, mental health, substance use disorder, addictive behavior

## Abstract

The most important practical concerns in addiction medicine are the non-substance addiction and related addictive behaviors among individuals with substance use disorder. On the other hand, technological advances, and easy access have increased the frequency of online sexual activities (OSAs) as one of these behaviors. This study aimed to compare the prevalence of OSAs, based on the Internet Sex Screening Test (ISST) scores, among 60 patients with substance use disorder referred to Iran Psychiatric Hospital and 60 non-dependent individuals. The results showed significant negative correlations between the ISST scores and age, age at the onset of substance use, and substance use duration. There was a significant difference between the ISST scores of the case and control groups (*P* = 0.001). Patients who start using substances at an early age and have a great duration of substance use are more likely to engage in other addictive behaviors such as OSAs. Therefore, it is critical to consider OSAs and other addictive behaviors in patients with substance use disorder to provide better care for this vulnerable community.

## Introduction

Addiction is a chronic recurrent psychiatric disorder in which a person has a psychological and physiological need to use a substance and shows withdrawal symptoms if it is discontinued. Many studies have shown that substance use disorder is often associated with other major psychiatric disorders such as anxiety and mood disorders (1). But the new definition of addiction in the Diagnostic and Statistical Manual of Mental Disorders, 5th Edition (DSM-5) is not limited to the use of substances (2). And different addictive behaviors have been clustered like sex addiction, gambling addiction, and internet addiction. Internet addiction can manifest as addiction to online shopping, online computer games, cyberspace, social media, and online pornography. It brings more concerns for clinicians, patients, and their caregivers and needs to be addressed in a clinical context (3). Addictive behaviors are the unexplored area of research in which the co-occurrence of substance use disorder and other addictive behaviors and their problematic consequences in societies nowadays are discussed in different consensus (4).

Addiction is one of the leading health problems in Iran these days, and many studies have been done on different aspects of addiction in the last decades. Still, there are lack of robust scientific data on different addictive behaviors (5). Addictive behavior activates the reward and pleasure system in individuals. Recent research showed the underlying etiology of all types of addiction is the reward system and the mesolimbic dopaminergic pathway.

The Reward system provides a sense of reward and accomplishment; it encourages individuals to engage in healthy behaviors and regulates motivation and emotion. Repetitive exposure to stimuli like substances or other addictive activities can cause dysregulation at molecular and cellular levels. This dopamine dysregulation and dysfunctional reward systems have been found in all types of addiction. Severe consequences of dysregulation in dopamine and reward circuits are major public mental health issues (6).

Due to the brain circuit complexity, destructive behaviors are perceived as a method to escape discomforts and inconveniences and fail to reduce the urge and craving. This dysregulation in the choice and loss of control will lead to a pathological pattern of use, neglect of personal and professional aspects of life, and devastating consequences in societies (7).

Online sex addiction is one of the latest types of online addictive behaviors. Various factors such as technological and innovative advancements, user-friendly, inexpensive, anonymous access through millions of websites available 24/7, the absence of the risk of a sexually transmitted disease (STD) and risk of abortion, less physical trauma, psychosexual dissatisfaction, and relationships without commitment, have increased the prevalence of online sex addiction. Online sex addiction (OSA) is defined as a set of compulsive online sexual activities (OSAs), e.g., watching online pornography, downloading videos with sexual content, engaging in erotic chats and sharing one's most profound sexual fantasies with others, online role-playing based on another person's sexual fantasies, performed primarily for pleasure. However, over time, this sense of purpose and pleasure is replaced by self-destruction, decreased control over sexual behaviors, and a strong desire to continue the activity (8, 9).

According to Young, some warning signs such as spending notable amounts of time on the internet for cybersex, preoccupations with sexual content while using the internet, feeling guilt or shame about using the internet for sexual purposes, masturbating while using the internet, and engaging in erotic chat rooms, and preference of cybersex as the primary form of sexual gratification, can indicate the presence of online sex addiction (10). Online sex addiction can have devastating and irreversible consequences, including feeling tired, ashamed, remorseful, lonely, disappointed in not having a healthy sex life, frustrated with time-wasting, and absent from work (11, 12). A U.S. survey found that 13% of internet searches contained sexual terms, 35% of internet downloads were related to pornography, and 30.6% of users engaged in sexual conversations with other people online (13).

From the perspective of Iranian culture, family formation is very blessed, family formation is an integral part of people's lives, and marriage is the only official way to have sexual activities (8). On the other hand, many people cannot start a family at a young age due to socio-economic issues. According to society's cultural, traditional model, young individuals often live with their family members until marriage. Sometimes they have a financial dependency and emotional attachment to their family.

Many studies have shown that one of the leading causes of divorce in society is sexual issues, and the lack of formal education and proper sexual knowledge and lack of sufficient skills in marriage may lead to curiosity in people to learn sex education through informal methods and sexual use of the Internet (14). Also, like many other underlying causes of people's tendency to addictive behaviors, maladaptive coping styles and lack of an efficient system to control impulses against failures, anger, anxiety, and stress in life, can be the underlying causes of online sex addiction (15). The comorbidity of anxiety and mood disorders and substance use has been shown to have a higher prevalence among people involved in online sex addiction (16). Highlighting the co-occurrence of other addictive behaviors with substance use disorders is crucial to explore more about biology, epigenetic, and environmental factors underlying different addictive behaviors that help clinicians to identify new strategies for prevention management and novel treatment interventions. A limited number of studies have evaluated the prevalence of OSA among individuals with substance use disorder. Their findings have shown that online sex addiction is more prevalent among the mentioned group (17, 18). To the best of our knowledge, no previous study has investigated OSAs in Iran patients with substance use disorder. Therefore, this study compared the prevalence of OSAs among Iranian people with and without substance use disorder.

## Methods

### Design and Participants

This case-control study was conducted at Iran Psychiatric Hospital, affiliated with the Iran University of Medical Sciences, in 2020. The research population included 60 individuals with substance use disorder and 60 persons in the control group (a total of 120). The participants were selected by non-random sampling. All persons referred to substance use clinics were interviewed, and those with the research inclusion criteria were recruited. The inclusion criteria for the case group were 18–50-year-old individuals with substance use disorder diagnosis, regular referral to the clinic in the past 6 months, ability to read, write, and use the internet, and informed consent for participation. The controls were selected to match the case group in terms of gender and age. The exclusion criteria were major psychiatric disorders, visual and hearing impairment, lack of fluency in Persian language, and intoxicated patients or patients with withdrawal symptoms. People who could not ensure constant participation in the study were also excluded.

### Instruments

#### Demographic Questionnaire

All demographic information was collected through questionnaires developed by the researchers.

#### Internet Sex Screening Test

The ISST was designed by Delmonico et al. in 1997. It is a self-administered screening instrument for individuals with online sex addiction (19). The Persian version of this test has 20 true/false items arranged in a five-factor structure. These factors include online sexual compulsivity, online sexual behavior-social, online sexual behavior-isolated, online sexual spending, and interest in online sexual behavior. Cronbach's alpha for the subscales range between 0.51 and 0.86. In 2019, Shalbafan et al. evaluated the validity and reliability of the Persian version of the test and reported Cronbach's alpha values for the subscales as 0.7–0.9 (20).

### Ethics Approval and Consent to Participate

All principles of the Declaration of Helsinki were followed. This study was also approved by the Ethical Review Board of Iran University of Medical Sciences, Tehran, Iran (NO.: IR.IUMS.FMD.REC.1399.274). In this study, written consent was obtained from all patients, and the names and details of the patients were not used in the study.

## Results

A total of 120 individuals were allocated to a case group (diagnosed with substance use disorder; *n* = 60) and a control group (healthy individuals; *n* = 60). The characteristics of the participants are summarized in Table 1.

**Table 1:** Characteristics of the study sample (*n* = 120).

**Variables**	**Case group**		**Control group**		***P***
**Variables**	**(*****n*** **=** **60)**		**(*****n*** **=** **60)**		
	**N/M**	**%/SD**	**N/M**	**%/SD**	
Gender					0.889
Male	51	85	50	83.3	
Female	9	15	10	16.7	
Marital status					0.049
Single	24	40	34	56.7	
Married	21	35	18	30	
In a relationship	4	6.7	7	11.6	
Divorced	11	18.3	1	1.7	
Level of education					<0.001
High school	11	18.3	0	0	
Diploma	24	40	4	6.7	
Academic	25	41.7	56	93.3	
Age (Years)	35.13	8.14	28.97	7.37	0.154
Age at the onset of substance use (Years)	22.70	5.31	NA	NA	-
Duration of substance use (Years)	11.45	8.15	NA	NA	-
Internet sex screening test score^*^	3	3.96	1.17	0.37	0.001

* *Cohen's d = 0.650704*.

Opium was the most widely used substance in the case group (*n* = 39; 65%). Independent *t*-test results showed a significant difference between the two groups in terms of ISST scores (*P* = 0.001; df = 118; *t* = 3.56). Furthermore, the Pearson correlation coefficients showed that the ISST scores had a significant negative correlation with age (*P* < 0.001, *r* = −526), onset of substance use (*P* = 0.029, *r* = −283), and duration of substance use (*P* = 0.003, *r* = −378). There were no significant relationships between ISST scores and gender, marital status, and education level in the case or the control group.

## Discussion

The present study aimed to compare OSAs (based on ISST scores) in individuals with and without substance use disorder. The present study had a significant relationship between OSAs and substance use disorder. The results showed significant negative correlations between ISST scores and the age, age at the onset of substance use, and substance use duration. There was no significant correlation between ISST scores and gender, marital status, and education level in the case or the control group. The most commonly used substance was opium (65%). The high prevalence of OSAs was consistent with a study conducted in 2012 to investigate the rate of internet sex addiction (11). Likewise, a systematic review study in 2020 showed that OSAs were more common among people with substance use disorder (17). In 2018, Bosma et al. examined sexual behaviors among substance users. They used a pre-coded questionnaire designed by the researchers. Among 180 patients with substance use disorder, 31.6% mentioned a previous history of using pornography (18).

Moreover, in 2000, Schwartz et al. evaluated obsessive-compulsive behaviors in people with internet sex addiction. Similar to our findings, they detected a higher prevalence of substance use disorder in people with online sex addiction. They found that 73.7% of men and 42.9% of women with online sex addiction used substances, particularly cocaine and alcohol and drugs such as benzodiazepines (21). Similarly, in 2017, Morelli et al. studied internet addictive behaviors among adults and detected a significant association between internet addiction and high-risk sexual behaviors, alcohol consumption, and substance use disorder (22).

Consistent with our study, Najavits L et al. evaluated the co-occurrence of addictive behaviors in patients with substance use disorder. Addictive behaviors like spending money addiction; sex or pornography addiction; work addiction; exercise addiction; self-harm addiction; computer or internet addiction; eating addiction to alcohol and opium users were predominant, and pornography and internet addiction was greater among younger male adults (23). In 2013, Sik Lee et al. investigated the prevalence of substance use disorder and sex addiction and the likelihood of an association between the two behaviors. The study included a large population of 13–18-year-old individuals and found that 85.2% of the participants were public users. In addition, 9.11% of the subjects were at potential risk of internet addiction, and 3.0% were high-risk. Substance use disorder differed in these three groups and was 1.7, 2.0, and 6.5%, respectively (24). Despite the differences in participants' age, which makes comparing their results and ours difficult, their reports emphasize the importance of considering OSAs from earlier generations.

In 2015, Weinstein et al. Evaluated the cybersex use predictive factors among male and female users. Consistent with our study, the male users were dominant. They reported that craving for pornography and relationship challenges were the predictive factors for the frequency of using online sex activities (25).

According to the findings of this study and previous studies, from the psychiatric viewpoint, there is a correlation between various pathological behaviors in the field of psychiatry, especially in behavioral addiction; unless they are identified and addressed, complete treatment of a disorder alone will not lead to full remission. A comprehensive look at the patient and the nature of the patient's behavior during their life frame is needed to prevent relapse, which causes distrust and compassion fatigue for the patients and their families (26).

The attention of addiction therapists to the correlation between addictive behaviors and the various comorbidities that exist between them and other major psychiatric disorders can be critical. Using effective pharmacological and non-pharmacological therapies such as cognitive-behavioral therapy, relapse prevention, and motivational interview programs, focusing on the maladaptive cognitions, and improving their social support network can help these patients and reduce the burden of addictive behaviors on the health care system.

### Limitations

Our study had several limitations. First, its statistical population was small, and also, the subjects that were referred to the clinic where we collected our subjects were mostly men subjects. The gender distribution of our subjects was affected. Moreover, it was impossible to maintain gender balance as substance users are generally men. Further studies are recommended to discuss the reasons and factors that cause the gender difference in OSA, such as personality traits, psychosocial factors, more social stigma in women's addiction issues, different help-seeking behavior patterns, etc. (27). Since the tool used in this study does not have the power to report subtypes of online sexual activity, it is recommended to increase the sample size, use more female participants, and report the different subtypes of online sexual activity in future studies.

On the other hand, due to the cultural characteristics of the Iranian society, low participation rate, and the possibility of incorrect responses (considering the social stigma caused by the issue), there might be a gap between the obtained results and the existing reality. Social stigma leads to adverse social, psychological, and psychical outcomes. Fear and shame produced due to stigma decrease treatment-seeking behaviors, and the families may recommend substance users discontinue treatment to avoid the consequences of stigma. Further studies are needed to be done to evaluate the different online sexual sub-activities such as watching or downloading porn, engaging in erotic chats, etc., and show the correlation of each activity with gender, ethnicity, age, educational level, and other addictive behaviors.

### Implications for Practice and Research

Based on our findings, OSAs are more prevalent among Iranian patients with substance disorders than individuals without substance use disorders. This calls for more careful attention for mental healthcare providers to explore deeply other addictive behaviors other than substance use. It should be noted that this attention is more important among traditional religious, particularly in Islamic societies like Iran, where involvement in any extramarital sexual activities like OSAs may have a lot of problematic consequences in various dimensions of life.

In recent years, as globalization has expanded and the use of cyberspace and online communications has risen, developing societies like Iran have shifted from traditional to modern societies, and individuals' definitions of the family construct and sexual activities in the context of marriage have changed (8). Due to this concern from patients, most clients probably do not report engagement in these activities spontaneously, even when they are distressing and problematic. Therefore, a healthcare worker needs to approach these activities in the history of the patients. However, the confidentiality of documentation of this issue should be carefully considered to protect the patient from unfavorable consequences.

A larger sample size and a more extended female target population will be required in future studies on the relationship between addictive behaviors such as OSAs and other psychiatric disorders. The factors involved in exacerbating OSAs and substance use disorder should also be identified to facilitate the formation of appropriate executive solutions and ultimately control behavioral addictions and increase the quality of life of people.

## Conclusions

According to our findings, OSAs are more prevalent among Iranian patients with substance disorders than individuals without substance use disorders. In addition, individuals who start substance use at an early age and have a long history of substance use are more likely to engage in other addictive behaviors such as OSAs. Therefore, it is critical to consider OSAs and other addictive behaviors in patients with substance use disorder to provide better care for this vulnerable community. Further investigations by multi-center studies with a larger sample are needed to determine related factors.

## Data Availability Statement

The raw data supporting the conclusions of this article will be made available by the authors, without undue reservation.

## Ethics Statement

The studies involving human participants were reviewed and approved by the Ethical Review Board of Iran University of Medical Sciences, Tehran, Iran (Code: IR.IUMS.FMD.REC.1399.274). The patients/participants provided their written informed consent to participate in this study.

## Author Contributions

SS, VR, FH, HA, and MSh: conceptualization and design. SS, PH, and MSh: data collection. SS, MSa, and MSh: initial draft preparation. SS, VR, MSa, PH, FH, HA, and MSh: editing and review. All authors contributed to the article and approved the submitted version.

## Conflict of Interest

The authors declare that the research was conducted in the absence of any commercial or financial relationships that could be construed as a potential conflict of interest.

## Publisher's Note

All claims expressed in this article are solely those of the authors and do not necessarily represent those of their affiliated organizations, or those of the publisher, the editors and the reviewers. Any product that may be evaluated in this article, or claim that may be made by its manufacturer, is not guaranteed or endorsed by the publisher.

## References

[1] Soraya S Mahdavi M Saeidi M Seddigh R Nooraeen S Sadri M. Prevalence of anxiety disorders and its co-occurrence with substance use disorder: a clinical study. Middle East Curr Psychiatry. (2022) 29:30. 10.1186/s43045-022-00197-x

[2] American Psychiatric Association. Diagnostic and statistical manual of mental disorders (DSM-5®): American Psychiatric Pub. (2013). 10.1176/appi.books.97808904255968723190

[3] Thombs DL. An Introduction to Addictive Behaviors, 2nd Edn. New York, NY: Guilford Press (1999).

[4] De Alarcón R de la Iglesia JI Casado NM Montejo AL. Online porn addiction: what we know and what we don't—A systematic review. J Clin Med. (2019). 8:91 10.3390/jcm8010091 30650522 PMC6352245

[5] Roche GC Fung P Ransing R Noor IM Shalbafan M El Hayek S. The state of psychiatric research in the Asia Pacific region. Asia Pac Psychiatry. (2021) 13:e12432. 10.1111/appy.12432 33145988

[6] Baik JH. Dopamine signaling in reward-related behaviors. Front Neural Circuits. (2013). 7:152. 10.3389/fncir.2013.00152 24130517 PMC3795306

[7] Lapointe L Boudreau-Pinsonneault C Vaghefi I editors. Is Smartphone Usage Truly Smart? A Qualitative Investigation of IT Addictive Behaviors. 46th Hawaii international conference on system sciences. IEEE (2013). 10.1109/HICSS.2013.367

[8] Mokhtari S Shariat SV Ardebili ME Shalbafan M. Iranian students' attitudes toward premarital sex, marriage, and family in different college majors. J Am Coll Health. (2022) 70:1186–94. 10.1080/07448481.2020.1789150 32672512

[9] Schimmenti A Passanisi A Gervasi AM Manzella S Famà FI. Insecure attachment attitudes in the onset of problematic Internet use among late adolescents. Child Psychiatry Hum Dev. (2014) 45:588–95. 10.1007/s10578-013-0428-0 24338269

[10] Young KS. Internet sex addiction: risk factors, stages of development, and treatment. Am Behav Sci. (2008) 52:21–37. 10.1177/000276420832133935793576

[11] Griffiths MD. Internet sex addiction: a review of empirical research. Addict Res Theory. (2012) 20:111–24. 10.3109/16066359.2011.588351

[12] Grubbs JB Volk F Exline JJ Pargament KI. Internet pornography use: perceived addiction, psychological distress, and the validation of a brief measure. J Sex Marital Ther. (2015) 41:83–106. 10.1080/0092623X.2013.842192 24341869

[13] Albright JM. Sex in America online: an exploration of sex, marital status, and sexual identity in Internet sex seeking and its impacts. J Sex Res. (2008) 45:175–86. 10.1080/00224490801987481 18569538

[14] Maasoumi R Lamyian M Khalaj Abadi Farahani F Montazeri A. Women's perception of sexual socialization in Iran: a qualitative study. J Qual Res Health Sci. (2020) 2:221–33.

[15] Moshtagh M Rafiey H Mirlashari J Azin A Farnam R. Facilitators of and barriers to compulsive sexual behavior in Iranian women. Sex Addict Compulsivity. (2017) 24:270–84. 10.1080/10720162.2017.1358124

[16] Berberovic D. Sexual compulsivity comorbidity with depression, anxiety, and substance use in students from Serbia and Bosnia and Herzegovina. Eur J Psychol. (2013) 9:517–30. 10.5964/ejop.v9i3.595

[17] Hermand M Benyamina A Donnadieu-Rigole H Petillion A Amirouche A Roméo B. Addictive use of online sexual activities and its comorbidities: a systematic review. Curr Addict Rep. (2020) 7:194–209. 10.1007/s40429-020-00301-3

[18] Bosma-Bleeker MH Blaauw E. Substance use disorders and sexual behavior; the effects of alcohol and drugs on patients' sexual thoughts, feelings and behavior. Addict Behav. (2018) 87:231–7. 10.1016/j.addbeh.2018.07.005 30077915

[19] Delmonico DL. Internet Sex Screening Test. (1997). Available online at: http://www.sexhelp.com. 10.1037/t60529-000

[20] Shalbafan M Najarzadegan M Soraya S Rashedi V Najafian R Ahmadkhaniha H. Validity and reliability of the Persian version of Internet Sex Screening Test in Iranian medical students. Sex Addict Compulsivity. (2019) 26:361–70. 10.1080/10720162.2019.1645060

[21] Schwartz MF Southern S. Compulsive cybersex: the new tea room. Sex Addict Compulsivity. (2000) 7:127–44. 10.1080/10720160008400211

[22] Morelli M Bianchi D Baiocco R Pezzuti L Chirumbolo A. Sexting behaviors and cyber pornography addiction among adolescents: the moderating role of alcohol consumption. Sex Res Soc Policy. (2017) 14:113–21. 10.1007/s13178-016-0234-0

[23] Najavits L Lung J Froias A Paull N Bailey G. A study of multiple behavioral addictions in a substance abuse sample. Subst Use Misuse. (2014) 49:479–84. 10.3109/10826084.2013.858168 24304172

[24] Lee YS Han DH Kim SM Renshaw PF. Substance abuse precedes internet addiction. Addict Behav. (2013) 38:2022–5. 10.1016/j.addbeh.2012.12.024 23384457 PMC4651446

[25] Weinstein AM Zolek R Babkin A Cohen K Lejoyeux M. Factors predicting cybersex use and difficulties in forming intimate relationships among male and female users of cybersex. Front Psychiatry. (2015) 6:54. 10.3389/fpsyt.2015.00054 25941496 PMC4403291

[26] Alavi SS Ferdosi M Jannatifard F Eslami M Alaghemandan H Setare M. Behavioral addiction versus substance addiction: correspondence of psychiatric and psychological views. Int J Prev Med. (2012) 3:290–4. 22624087 PMC3354400

[27] Shimoni L Dayan M Cohen K Weinstein A. The contribution of personality factors and gender to ratings of sex addiction among men and women who use the Internet for sex purpose. J Behave Addict. (2018) 7:1015–21. 10.1556/2006.7.2018.101 30378460 PMC6376399

